# A COMPREHENSIVE DIGITAL SELF-CARE SUPPORT SYSTEM FOR OLDER ADULTS IN PAIN: IMPACT OF SOCIAL SUPPORT ON CHRONIC PAIN

**DOI:** 10.1093/geroni/igae098.2426

**Published:** 2024-12-31

**Authors:** Priya Nambisan, Julie Ellis, Murad Taani, Yura Lee, Meenakshi Anupindi, Rose Nortey

**Affiliations:** University of Wisconsin Milwaukee, Milwaukee, Wisconsin, United States; University of Wisconsin Milwaukee, Milwaukee, Wisconsin, United States; University of Wisconsin Milwaukee, Milwaukee, Wisconsin, United States; University of Wisconsin-Milwaukee, Milwaukee, Wisconsin, United States; University of Wisconsin Milwaukee, Milwaukee, Wisconsin, United States; University of Wisconsin Milwaukee, Milwaukee, Wisconsin, United States

## Abstract

Social support has been associated with positive health outcomes among older adults, contrasting with the adverse effects associated with social isolation and loneliness, which are linked to poor physical and mental health. Previous studies examined social isolation, loneliness, and physical health either at different time points or through correlations within secondary data. While interventions incorporating social support have demonstrated improvements in health and alleviation of symptoms such as pain for older adults, comprehensive assessments require controlling for various factors such as the impact of medication, diet, exercise, and other activities. Remarkably, observational studies simultaneously measuring social support, physical health changes, and factors like physical activity and diet are lacking. This pilot study uses a Comprehensive Digital Self-Care Support System (CDSSS), equipped with tools to monitor over 50 physical and mental health conditions, diet, activity, sleep, medication intake, alcohol use, smoking, and other self-care activities. The CDSSS also includes a robust online forum with video chatting facilities. This mixed methods pilot study aims to test whether we can evaluate in real time the changes in health indicators such as stress, mood, and chronic pain when participants engage in the online social space within the CDSSS. Participants are Black older adults (age >50), suffering from chronic conditions where pain is the main symptom such as Migraines, Fibromyalgia, Arthritis, Gout, and Backpain. This study integrates qualitative data from the social support forum and interviews, along with quantitative data from over 13 pain-specific trackers and surveys measuring social support, loneliness and social isolation.

